# Surgery

**Published:** 1907-09-07

**Authors:** 


### SURGERY.

"The Manual of Surgery," by Charles Stonham, published by Messrs. Macmillan and Co., in three vols. (A thoroughly practical book containing in a readable form and in moderate compass all that a student need know about surgery while he is acting as a dresser.)

Walsham's "Theory and Practice of Surgery," edited by W. G. Spencer, published by Messrs. Churchill. (Contains a very large amount of information in comparatively few pages, and for this reason it is a little difficult to read in at all a dilettante fashion.)

Rose and Carless' "Manual of Surgery," published by Messrs. Baillière and Tindall. (This text-book runs neck and neck with the preceding in popular favour. Its format is perhaps a little more pleasing than that of its rival.)

Cheyne and Burghard's "Manual of Surgical Treatment," in six parts, Messrs. Longmans, Green, and Co. (Very complete, thoroughly practical, and in every way satisfactory for a senior student.)

Jacobson and Steward's "Operations of Surgery," published by Messrs. Churchill, in two volumes. (Perhaps the most satisfactory English text-book of operative surgery extant. The fifth is now in course of preparation.)

"A System of Practical Surgery," edited by Profs. Von Bergmann, Bruns and Mikulicz, translated into English and edited by Drs. Bull, Frost, Flint, and Martin, issued by Messrs. Williams and Norgate in four vols. (This system gives a very complete account of the science and art of surgery at the present time.)

"A System of Surgery," edited by Sir Frederick Treves, in two volumes, published by Messrs. Cassell and Co. (The work consists of an excellent series of articles by surgeons attached to the various London hospitals, without any attempt to make it encyclopædic. It may be supplemented by Sir Frederick Treves' Manual of Operative Surgery, which is also published by Messrs. Cassell, and in two volumes.

Surgical anatomy and surgical pathology form the backbone of surgery as a science. But too many students are content to learn only so much as is sufficient to enable them to obtain a minimum examination knowledge. There are several good English text-books on surgical anatomy. One of the best is certainly M'Lachlan's "Applied Anatomy: Surgical, Medical and Operative," in two volumes, published at Edinburgh, by Messrs. E. and S. Livingstone. It is clear, well written and well illustrated.

Sheild's "Surgical Anatomy," published by Mr. Young J. Pentland, is a smaller work without illustrations, and is meant to be used with the living model. Rawling's "Landmarks and Surface Markings of the Human Body," published by H. K. Lewis, is somewhat on the lines of the late Mr. Holden's "Medical and Surgical Landmarks." Mr. Rawling's book is well illustrated, and has already reached a second edition.

Leaf's "Surgical Anatomy of the Lymphatic Glands," published by Messrs. Archibald Constable and Co., is of greater service to the surgeon than the student.

Sir Frederick Treves' "Surgical Applied Anatomy" has long been a favourite with students. A revised edition has recently been issued by Dr. Arthur Keith, and published by Messrs. Cassell and Co. It is in one volume well illustrated.

Messrs. Sherratt and Hughes, of Manchester, issue a revised edition of "A Handbook of Surgical Anatomy," by Professor G. A. Wright and Dr. C. H. Preston.

Messrs. Box and McAdam Eccles have recently published, through Messrs. Churchill, a book on "Clinical Applied Anatomy," which is in many respects an interesting volume, reminding the reader a little of Hilton's "Rest and Pain," which is so well known to every thoughtful student of surgery. It indicates the important influence of anatomy on the incidence and progress of disease, disorder and injury of the human body.

Surgical pathology can only be learnt in the post-mortem room and the museum. No attempt should be made to learn it by reading. The text-book should be taken into the museum and the description which it contains ought to be verified by the different specimens to be found in every well-arranged collection. Mr. Bowlby's "Surgical Pathology" (Messrs. J. and A. Churchill) contains a large number of drawings, and describes in simple language the various pathological processes which are interesting to the student of surgery.

Walsham's "Surgical Pathology" is a catalogue *raisonnée* of the Museum at St. Bartholomew's Hospital. The third edition, published by Mr. Paterson, has been adapted for use in pathological collections other than that for which it was originally written.

Buchannan's (A. M.) "Manual of Anatomy" (Baillière, Tindall, and Cox) is in two parts, each 12s. 6d., and forms an excellent work for ordinary students or for honours examinations.

Messrs. Ashby and Wright's "Diseases of Children" (Messrs. Longmans, Green and Co.) is convenient because it contains the medical and surgical diseases in a single volume.

Mr. Edmund Owen's "Surgical Diseases of Children" (Messrs. Cassell and Co.) and Mr. D'Arcy Power's "Surgical Diseases of Children and their Treatment by Modern Methods" (Mr. H. K. Lewis), are well illustrated, and give the views of experienced surgeons who have been attached for many years to large children's hospitals.

"Deformities: A Treatise on Orthopædic Surgery," by Mr. A. H. Tubby (Messrs. Macmillan and Co.) is certainly the best and most complete text-book by an English surgeon in this department of surgery. It may be supplemented by "The Surgery of Paralyses," by Messrs. Tubby and Robert Jones.

"Orthopædic Surgery: A Text-book of the Pathology and Treatment of Deformities," by Mr. Jackson Clarke (Messrs. Cassell and Co.) is a successful attempt to give an outline of the various therapeutic measures applicable to deformities, the treatment being based upon the pathology of the condition.

"Diseases of the Nose and Throat," by Dr. De Havilland Hall and Mr. Herbert Tilley, fully maintains the reputation of Mr. H. K. Lewis' practical series. It forms an easy introduction to a special and important branch of practice which is too often neglected by students because of the slight initial difficulty of learning to use the laryngoscope.

"Diseases of the Throat, Nose and Ear," by Dr. McBride (Young J. Pentland) conveys within a volume of reasonable dimensions the main facts of modern laryngology, rhinology and otology.

Mr. Mark Hovell's "Treatise on Diseases of the Ear and Naso-Pharynx" (Messrs. J. and A. Churchill) gives a very thorough account of aural surgery, and of affections of the nose and pharynx.

Dr. William Lamb's "Guide to the Examination of the Throat, Nose and Ear" (Baillière, Tindall, and Cox, 5s.) is one of the most valuable books of the kind. It is practical, and suitable to all workers in that speciality.

Dr. Wyatt Wingrave's "Adenoids" (Baillière, Tindall, and Cox, 2s. 6d.) is an exhaustive and essentially practical account of this condition, sound in views and a safe guide, and can be confidently recommended.

Dr. James B. Ball's "Handbook of the Diseases of the Nose and Pharynx" (Baillière, Tindall, and Cox, 7s. 6d.) devotes special attention to the question of treatment.

Roosa and Douglas. "Diseases of Ear, Throat, and Pharynx." Macmillan. 12s. 6d. net.

Dr. Love's "Diseases of the Ear," 25s. (John Wright and Co., Bristol).

